# Interplay Effect of Target Motion and Pencil-Beam Scanning in Proton Therapy for Pediatric Patients

**DOI:** 10.14338/IJPT-17-00030.1

**Published:** 2018-11-30

**Authors:** Andrew J. Boria, Jinsoo Uh, Fakhriddin Pirlepesov, James C. Stuckey, Marian Axente, Melissa A. Gargone, Chia-ho Hua

**Affiliations:** 1 Department of Radiation Oncology, St. Jude Children's Research Hospital, Memphis, TN, USA; 2 School of Health Sciences, Purdue University, West Lafayette, IN, USA; 3 Department of Physics, Rhodes College, Memphis, TN, USA

**Keywords:** pediatric patients, proton therapy, particle therapy, tumor motion, interplay effect

## Abstract

**Purpose:**

To investigate the effect of interplay between spot-scanning proton beams and respiration-induced tumor motion on internal target volume coverage for pediatric patients.

**Materials and Methods:**

Photon treatments for 10 children with representative tumor motions (1–13 mm superior-inferior) were replanned to simulate single-field uniform dose–optimized proton therapy. Static plans were designed by using average computed tomography (CT) data sets created from 4D CT data to obtain nominal dose distributions. The motion interplay effect was simulated by assigning each spot in the static plan delivery sequence to 1 of 10 respiratory-phase CTs, using the actual patient breathing trace and specifications of a synchrotron-based proton system. Dose distributions for individual phases were deformed onto the space of the average CT and summed to produce the accumulated dose distribution, whose dose-volume histogram was compared with the one from the static plan.

**Results:**

Tumor motion had minimal impact on the internal target volume hot spot (D2), which deviated by <3% from the nominal values of the static plans. The cold spot (D98) was also minimally affected, except in 2 patients with diaphragmatic tumor motion exceeding 10 mm. The impact on tumor coverage was more pronounced with respect to the V99 rather than the V95. Decreases of 10% to 49% in the V99 occurred in multiple patients for whom the beam paths traversed the lung-diaphragm interface and were, therefore, more sensitive to respiration-induced changes in the water equivalent path length. Fractionation alone apparently did not mitigate the interplay effect beyond 6 fractions.

**Conclusion:**

The interplay effect is not a concern when delivering scanning proton beams to younger pediatric patients with tumors located in the retroperitoneal space and tumor motion of <5 mm. Children and adolescents with diaphragmatic tumor motion exceeding 10 mm require special attention, because significant declines in target coverage and dose homogeneity were seen in simulated treatments of such patients.

## Introduction

Proton therapy is one of the most advanced modalities for treating cancer. Its primary advantage over conventional photon therapy is its ability to deliver a high radiation dose to a specified depth by exploiting the Bragg peak effect, resulting in better sparing of nontargeted critical structures [1, 2]. This is particularly useful when treating pediatric patients with cancer because it reduces the risk of radiation-induced toxicity and secondary cancer [3]. As a result, proton therapy has become the preferred modality for treating many types of pediatric cancer at those institutions where it is available [2–4].

Pencil-beam scanning technology has been rapidly embraced by proton therapy centers to provide better dose sculpting on the target and further reduce the dose to normal tissues [5]. This method swiftly steers a narrowly focused proton beam of a specific energy via a pair of scanning magnets in the treatment nozzle, rather than delivering a passively scattered broad beam collimated by a brass aperture. The most widely adopted pencil-beam scanning technique is discrete spot scanning, which irradiates spots one by one in a grid pattern through a sequence of energy layers within the target, allowing for the precise irradiation of irregularly shaped tumors [6].

Despite the advantages of pencil-beam scanning, it is more susceptible than other delivery methods to respiration-induced tumor motion and changes in tissue stopping power along the beam paths. The beam delivery normally occurs over several respiratory cycles. In different phases of each respiratory cycle, variations in the patient anatomy and tumor location may be sufficient to cause spots to be deposited in locations away from the areas originally planned. Consequently, the delivered dose distribution, summing the contributions from all the spots, can deviate from that in the original treatment plan [7, 8]. This phenomenon is referred to as the *interplay effect*.

The interplay effect has been investigated in adult patients with cancer. Volumetric repainting or the use of larger beam spots reduced the interplay effect in the treatment of mediastinal lymphoma [7], and radiation fractionation reduced the interplay effect in the treatment of stage III lung cancer [9]. Four-dimensional proton treatment planning for lung tumors has also been found to be much more capable of delivering the prescribed dose during the respiratory cycle by comparison with planning based on a standard helical computed tomography (CT) data set, with the latter potentially failing to deliver up to 36% of the prescribed dose [10]. Strategies to mitigate the motion interplay effect, such as repainting, respiratory gating, and tumor tracking, have already been implemented clinically or investigated in adult patients [11–13].

To date, knowledge regarding the motion interplay effect in pediatric proton therapy has been extremely limited. This is probably due to the more urgent need to ensure safe delivery of proton therapy to adult patients with lung cancer or abdominal tumors. The limited number of pediatric patients with moving body tumors who receive proton therapy at any given institution also hinders such research. This study aimed to fill the knowledge gap by quantifying the interplay effect on the internal target volume (ITV) of pediatric patients with cancer who were treated with spot-scanning proton beams. Treatments were simulated with actual patient breathing traces of a respiratory cycle and the specifications of a recently installed synchrotron-based proton therapy system with reduced spot sizes (2–3 mm sigma at 210 MeV). We aimed to confirm that the plan quality could be preserved for pediatric patients in whom respiratory motion was small and to identify scenarios in which motion mitigation may become necessary.

## Materials and Methods

### Overview of Image Data

This retrospective study used image data obtained from pediatric patients who received photon radiation therapy directed to abdominal sites. As part of the treatment planning process, these patients underwent free-breathing 3D CT and 4D magnetic resonance imaging (MRI). No 4D CT was performed as it was desired to reduce the exposure of the children to radiation. The 4D MRI data were spatially registered to the 3D CT data to assist in delineating the ITV. In this study, for each patient, we generated a virtual 4D CT data set (also known as a “4D CT-MRI”), used this data set to plan a hypothetical proton treatment, and calculated the corresponding 4D dose distribution. The 4D CT-MRI technique was recently developed for mapping motion fields from 4D MRI data to a static 3D CT [14–16]. This technique enables 4D MRI to be used instead of 4D CT for 4D dose calculation and offers several advantages, including an absence of ionizing radiation, better detection of soft-tissue motion, and image orientation along the primary direction of respiratory motion.

### Patient Selection

**Figure 1:**
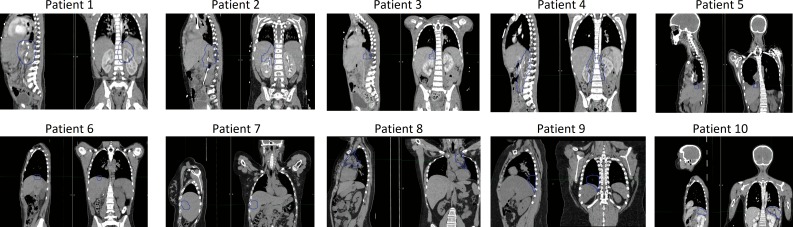
Sagittal and coronal computed tomography scans of the 10 patients with the internal target volume contours overlaid.

From a pool of 35 pediatric patients with abdominal tumors who underwent 4D MRI, we selected 10 representative patients who differed in age and in the extent of their tumor motion. **Table 1** lists the characteristics of the selected patients. The ITV locations are shown in **Figure 1**. Most of the younger patients who received 4D scans had neuroblastomas, and this is reflected in the patients selected for analysis. The retrospective use of the image data was approved by the Institutional Review Board.

**Table 1:** Patient characteristics.

**Patient No.**	**Sex**	**Age (y)**	**Height (cm)**	**Weight (kg)**	**ITV volume (cm^3^)**	**Respiration rate (breaths/min)**	**Maximum tumor motion^a^ (mm)**	**Diagnosis**
1	M	2	87	11.9	130.8	36.8	3.2	Neuroblastoma
2	F	4	100	17.6	117.6	34.4	1.2	Neuroblastoma
3	M	6	109	18.6	24.6	18.1	4.5	Neuroblastoma
4	M	9	127	19.9	295.8	29.5	4.8	Neuroblastoma
5	F	15	165	44	11.2	20.1	12.3	Osteosarcoma
6	M	15	172	56.3	21.7	17.8	7.9	Wilms tumor
7	F	18	168	79	67	19.3	9.4	Hodgkin lymphoma
8	F	19	160	65.4	111.7	17.2	6.2	Hodgkin lymphoma
9	M	15	157	92	87.4	24.9	9.2	Hodgkin lymphoma
10	M	11	143	32	176.2	21.5	13.5	Hodgkin lymphoma

**Abbreviations**: ITV, internal target volume; M, male; F, female.

a The maximum clinical tumor volume motion was the distance measured between 2 respiratory phases producing extreme tumor locations in the superior-inferior direction.

### 4D CT-MRI

The 4D MRI scan was performed on a Siemens MAGNETOM Avanto 1.5T MR scanner (Siemens Healthcare, Erlangen, Germany). Dynamic 2D images were acquired in coronal slices with an in-plane pixel size of 1.8 mm × 1.8 mm, a slice thickness of 4 mm, and a sampling rate of 3 Hz. The acquired images were sorted retrospectively, with an image-based respiratory surrogate being used to reconstruct image volumes at 10 respiratory phases [17]. The phases were temporally equidistant so that each phase corresponded to one-tenth of 1 respiratory cycle. The 3D CT was acquired on a Siemens SOMATOM Sensation Open scanner (Siemens Healthcare). The imaging parameters for CT were as follows: slice thickness of 1.52 mm, reconstructed pixel size of 0.65 to 0.98 mm, and tube voltage of 120 kVp.

The reconstructed 4D MRI was registered to the 3D CT by using MIM software (MIM Software Inc, Cleveland, Ohio). The image volume that was closest to the CT was visually identified, and a linear transformation was found by a semiautomatic procedure. The 4D MRI was also processed by a cyclic deformable registration, using elastix software [18, 19] to find motion fields between different respiratory phases. The motion fields were then linearly transferred to the 3D CT space to produce the 4D CT-MRI. Manual masking was applied to the transferred motion fields as needed in order to remove spurious respiratory motion caused by misregistration, image artifacts, or peristaltic/cardiac motion.

### Proton Treatment Planning

The proton treatments were planned by using an Eclipse Treatment Planning System (Varian Medical Systems, Inc, Palo Alto, California) and the specifications of our Hitachi PROBEAT-V synchrotron-based spot-scanning proton therapy system (Hitachi, Ltd, Tokyo, Japan). The proton convolution superposition dose calculation algorithm was used. The proton treatment plans in which the interplay effect was not simulated were based on the clinical photon treatment in terms of the prescribed dose and the number of fractions (**Table 2**). For patients who received multiple treatment courses directed at different targets, we selected the course that was most relevant to studying the effect of respiratory motion. An ITV was delineated by combining the clinical tumor volumes at all phases. The ITV was subsequently expanded by 5 mm to generate the planning target volume [9]. Two-field proton treatment was planned by using single-field optimization (also known as the single-field uniform dose) on a voxel-to-voxel average of the 4D CT-MRI (referred to as the average CT for simplicity) to achieve a uniform dose to the planning target volume in which the hot spots did not exceed 110% of the prescribed dose. The dose calculated on the average CT is referred to as the *nominal dose distribution* [7], because it does not take into account the interplay effect. The deformation field between the average CT and each phase of the 4D CT-MRI was found by using the aforementioned deformable registration tool to simulate the interplay effect.

**Table 2:** Summary of hypothetical proton treatment plans.

**Patient No.**	**Treatment site**	**No. of fractions**	**Dose per fraction (CGE)**	**Total dose (CGE)**	**Beam orientations**	**Field 1**		**Field 2**	
						**Gantry rotation (degree)**	**Couch rotation (degree)**	**Gantry rotation (degree)**	**Couch rotation (degree)**
1	Left adrenal	13	1.8	23.4	LL, PA	90	0	180	0
2	Right adrenal	13	1.8	23.4	PA, RAO	180	180	45	180
3	Right abdomen	13	1.8	23.4	RL, RPO	90	180	170	190
4	Right perineal	13	1.8	23.4	LPO, RL	145	0	90	180
5	Right diaphragm	8	5	40	RAO, RPO	65	180	115	180
6	Liver	6	1.8	10.8	RAO, RPO	55	190	100	190
7	Liver	17	1.5	25.5	RPO, PA	100	180	180	180
8	Mediastinum	17	1.5	25.5	LPO, RPO	150	0	150	180
9	Right lung base	17	1.5	25.5	LPO, RPO	150	0	150	180
10	Spleen	17	1.5	25.5	PA, LAO	180	180	20	0

**Abbreviations**: CGE, cobalt Gray equivalent; LL, left lateral; PA, posterior-anterior; RAO, right anterior oblique; RL, right lateral; RPO, right posterior oblique; LPO, left posterior oblique; LAO, left anterior oblique.

Note: All patients were planned in the supine position with 2 fields.

### Simulation of the Interplay Effect

The 4D dose distribution, taking into account the interplay effect, was calculated for comparison with the nominal dose distribution. The beginning of each treatment field at each fraction was randomly assigned to 1 of the 10 respiratory phases. The respiration rate was extracted from the respiratory surrogate signal that was used for reconstructing the 4D MRI, as seen in **Table 1**. The temporal information of the scanning spots was based on the machine specifications and log files from other patients who received actual proton treatments. The maximum duration of each acceleration cycle was 5 s. The interval between energy layers or spills for the deceleration and acceleration of protons was approximately 2 s. The time interval between adjacent spots was approximately 3 ms. The monitor units of each spot designated by the treatment plan were linearly converted to irradiation time by an empirical relation derived from the records of the dose monitor.

Once the respiratory phases of all the scanning spots had been determined, the accumulated dose distribution was calculated, including all spots delivered through a given respiratory phase, along with the corresponding CT-MRI volume at that phase. Then, the dose distributions for all phases were deformed onto the space of the average CT-MRI and combined. For the composite dose distribution of multiple fractions (which adjusts the fraction number and dose per fraction to maintain a total dose consistent with the patient's original photon therapy plan), this procedure was repeated according to the number of fractions (up to 25), with different random beginning phases and the resultant dose distributions being combined.

### Evaluation of the Interplay Effect

We calculated the dose to 98% and 2% of the ITV (D98 and D2, respectively) and the percentage of the ITV receiving at least 95% and 99% of the prescribed dose (the V95 and V99, respectively) in order to compare the 4D and nominal dose distributions. The beginning respiratory phase for each treatment field was randomly assigned 100 times. Average dosimetric parameters calculated from 100 runs are reported.

## Results

### Hot and Cold Spots

**Table 3** lists the average dosimetric parameters for 10 different patients and different numbers of fractions. In the original (static) plans, the hot spot (D2) was kept below 110% of the prescribed dose and the cold spot (D98) above 98.5% of the prescribed dose for all patients. In general, the motion interplay did not significantly increase the hot spot dose or decrease the cold spot dose by more than 3% for a given treatment fraction. The exceptions were patient 5 and 10, whose cold spot doses both became 15% lower than planned. Patient 5 was a teenager with a diaphragmatic tumor that had the second most extensive motion (12.3 mm) of any tumor in this study. For this patient, the effect observed in a single-fraction simulation did not improve when more fractions (up to 25) were added. Patient 10 was a child with a tumor involving the spleen that had the most extensive motion (13.5 mm) of any tumor in this study. Unlike for patient 5, the effect observed in a single-fraction simulation did improve to some extent when more fractions were added. For other patients, fractionation yielded marginal improvements in the hot and cold spots.

**Table 3:** Dosimetric evaluation of interplay effect.

**Dosimetric parameter**	**Patient**	**Original (static)**	**No. of fractions**						
			1	3	6	10	13	17	25
D2 (hot spot)	1	102.3	102.6	102.1	101.9	101.9	101.9	101.8	101.8
	2	104.5	104.1	104.1	104.0	104.0	104.0	104.0	104.0
	3	103.4	104.3	103.4	103.2	103.0	103.0	103.0	102.9
	4	103.2	104.0	103.0	102.8	102.8	102.7	102.7	102.7
	5	105.8	106.2	104.3	103.6	103.3	103.1	103.2	103.1
	6	110.0	109.6	109.1	109.0	109.0	108.9	108.9	108.9
	7	110.1	114.6	111.0	110.1	109.7	109.6	109.6	109.4
	8	109.3	111.7	109.5	108.8	108.5	108.4	108.3	108.2
	9	103.5	107.0	104.5	103.9	103.3	103.2	103.1	102.9
	10	109.6	113.8	107.4	106.4	105.6	105.1	104.7	104.2
D98 (cold spot)	1	99.1	97.7	98.1	98.3	98.4	98.4	98.4	98.4
	2	99.2	98.2	98.6	98.6	98.7	98.7	98.7	98.7
	3	99.4	97.3	98.1	98.3	98.4	98.4	98.4	98.5
	4	98.5	96.1	97.3	97.5	97.7	97.8	97.8	97.9
	5	100.0	85.8	86.0	86.3	86.5	86.5	86.5	86.5
	6	100.7	98.2	99.0	99.1	99.2	99.2	99.2	99.2
	7	101.6	93.8	97.8	99.2	99.8	100.2	100.4	100.7
	8	102.1	97.1	99.1	99.7	100.0	100.1	100.2	100.2
	9	100.3	95.3	97.7	98.4	98.9	99.0	99.2	99.4
	10	99.8	84.4	88.4	89.4	89.7	89.6	89.9	89.9
V95	1	100.0	100.0	100.0	100.0	100.0	100.0	100.0	100.0
	2	100.0	100.0	100.0	100.0	100.0	100.0	100.0	100.0
	3	100.0	99.9	100.0	100.0	100.0	100.0	100.0	100.0
	4	100.0	99.4	100.0	100.0	100.0	100.0	100.0	100.0
	5	100.0	80.4	80.8	81.7	81.9	81.9	82.1	82.2
	6	100.0	99.7	99.9	100.0	100.0	100.0	100.0	100.0
	7	100.0	96.9	99.5	99.9	100.0	100.0	100.0	100.0
	8	100.0	99.3	99.9	99.9	100.0	100.0	100.0	100.0
	9	100.0	98.3	99.9	100.0	100.0	100.0	100.0	100.0
	10	99.9	74.6	83.3	86.1	87.5	88.1	88.9	89.2
V99	1	98.4	83.4	87.1	88.6	89.1	89.2	89.5	89.8
	2	98.8	91.0	95.0	95.5	95.8	96.0	96.1	96.2
	3	99.2	86.8	92.5	94.4	95.2	95.7	95.7	96.1
	4	96.0	67.9	71.5	73.2	74.0	74.6	74.6	75.4
	5	99.4	55.4	58.3	60.0	60.4	60.4	60.6	60.9
	6	99.6	96.6	98.0	98.1	98.2	98.3	98.3	98.3
	7	100.0	85.3	95.2	97.9	98.8	99.4	99.5	99.7
	8	100.0	94.7	98.0	98.6	98.8	98.8	98.9	98.9
	9	100.0	79.2	90.5	94.2	97.4	97.5	98.3	99.2
	10	98.8	50.0	50.9	50.9	51.4	53.9	53.1	54.5
D5–D95	1	2.6	3.8	3.1	2.9	2.8	2.8	2.8	2.7
	2	3.9	4.4	4.0	3.9	3.9	3.9	3.9	3.9
	3	3.3	5.4	4.1	3.8	3.6	3.5	3.5	3.4
	4	3.5	6.1	4.4	4.0	3.8	3.8	3.7	3.6
	5	4.6	16.3	14.7	14.0	13.6	13.4	13.5	13.3
	6	7.5	9.0	8.0	7.9	7.8	7.8	7.8	7.8
	7	5.7	15.7	10.0	8.0	7.2	6.7	6.6	6.2
	8	5.5	11.0	7.7	6.7	6.1	6.0	5.8	5.7
	9	2.6	9.2	5.4	4.4	3.5	3.4	3.2	2.9
	10	7.7	22.8	14.5	12.8	11.9	11.6	11.0	10.7

### Target Coverage (V95 and V99)

**Figure 2:**
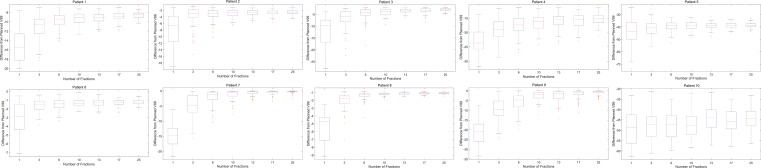
Differences between the accumulated V99s of the 4D treatments and the nominal V99s of the static plans. The box plots are displayed with red lines representing the median values; the top and bottom of each box representing the third and first quartiles, respectively; and the whiskers at the ends of the lines extending from the boxes representing the maximum and minimum, respectively (if there are no suspected outliers) or the positions corresponding to 1.5 times the difference between the third and first quartiles (the interquartile range) away from the box (if there are outliers). The red plus symbols represent potential outliers.

The target coverage of the static plans was quite high, except for patient 4, whose V99 was only 96%. This represented a compromise to reduce the doses to immediately adjacent critical structures. In a given fraction, the V95 was not sensitive to motion interplay, except in the case of patient 5. However, the V99 was substantially decreased (by 3%–48%) from that in the static plan for almost every patient. Patient 10 had the largest drop, but even the younger patients (patients 1–3, aged 2–6 years) who had relatively small tumor motion (1.2–4.5 mm) exhibited a change in V99 of up to 15%. When multiple treatment fractions were simulated, the V95 of the younger patients remained high and the V99 improved but never recovered to the planned values, even when treatment was simulated in 25 fractions. For older patients, fractionation yielded only limited improvements in V95 and V99 if a large interplay effect was already observed with 1 fraction, as was the case with patients 4, 5, and 10. Patient 9 was an exception to this and fractionation did yield noticeable improvements in specifically the V99. **Figure 2** illustrates how fractionation affected the V99 statistics. Note that the deviation from the nominal V99 for patients 4, 5, 9, and 10 remained large even with an increased number of treatment fractions.

### Inhomogeneity of the Target Dose

**Table 3** also shows the inhomogeneity of the dose to the ITV, defined as the D5–D95. For all patients except patient 5, the dose inhomogeneity converged to the planned values as the number of fractions increased. With 3 fractions, the deviation from the nominal values was already reduced to less than 1%. However, patient 5 still experienced an 8.7% increase in dose inhomogeneity, even when treatment was simulated in 25 fractions. Patient 10 had the highest dose inhomogeneity with 1 fraction (22.8%), although unlike patient 5, the dose inhomogeneity converged for this patient as the number of fractions increased.

## Discussion

In 4 patients with neuroblastoma who were younger than 10 years and whose tumors were located in the retroperitoneal space with a maximum vertical target motion of less than approximately 5 mm (patients 1–4 from **Table 1**), motion interplay caused minimal changes to the delivered dose. A V95 of 100% was achieved within 3 fractions for each patient. The hot-spot and cold-spot doses did not deviate significantly from the plans for any given fraction. The inhomogeneity of the target dose was within 0.5% of the original planned value in 6 fractions. The only dosimetric index analyzed that was sensitive to tumor motion was the V99. Thus, the interplay effect was largely negligible for younger patients with minimal superior-inferior target motion.

**Figure 3:**
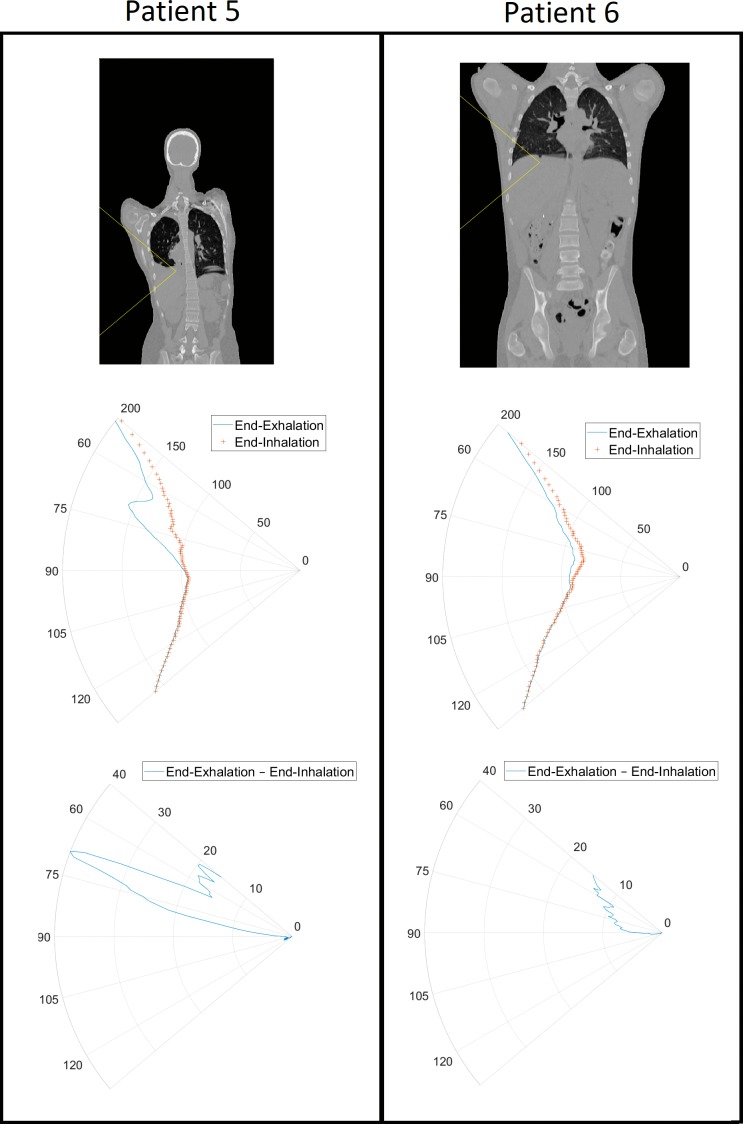
Water equivalent thickness (WET) as a function of gantry angle plots for patients 5 and 6 over the span of 50° to 130°. The top set of figures shows the angular distribution in a coronal slice of the body at the end of exhalation that the WET is being measured in. The middle set of figures shows the WET in millimeters as a function of angle at the end of exhalation and at the end of inhalation. The bottom set of figures shows the difference in WET in millimeters between the end of exhalation and the end of inhalation.

For adolescent patients in whom there was more extensive target motion, the interplay effects differed. Patients 5 and 6 were teenagers with tumors of similar sizes near the diaphragm. For patient 5, a significant interplay effect was manifested in the cold spot, tumor coverage, and dose inhomogeneity. However, patient 6 experienced no significant changes in the dosimetric indices examined. One major difference between these 2 patients was the extent of superior-inferior motion (12.3 mm for patient 5 versus 7.9 mm for patient 6). It was also easier to find beam angles with water-equivalent thickness changes that were less susceptible to respiration in patient 6 than in patient 5 as a result of the pathologic anatomy in the latter patient as can be seen in **Figure 3**. Expressing changes in water-equivalent thickness as a function of beam angle has been previously proposed [20–21]. Similar to what was observed for patient 6, tumor coverage of patients 7, 8, and 9 improved with increased fractionation. All 3 had tumor motion of less than 10 mm. Patient 10 had the greatest amount of tumor motion (13.5 mm) and exhibited the worst coverage loss, with similar V95 and V99 as seen in patient 5. This phenomenon of coverage loss is independent of the ITV volume, with patient 5 having the smallest ITV volume (11.2 cm^3^) while patient 10 has the second largest ITV volume (176.2 cm^3^). We hypothesize that children and adolescents with tumor motion of less than 10 mm can be safely treated with spot-scanning beams as long as the beam angles are properly chosen and the tumors are not particularly deeply situated, that is, the spot sizes are relatively large at shallower depths (energy layers). With a greater tumor motion than 10 mm, techniques such as respiratory gating and repainting should be considered.

Although the patients in this study were originally treated with photon therapy delivered in 6 to 17 fractions, up to 25 fractions of proton therapy were simulated to determine whether the interplay effect could be mitigated by using more fractions. It was noted that the V99 and the inhomogeneity indices improved as the number of simulated fractions increased from 1 to 6, whereas other indices remained fairly constant because they already approached the planned values. Fractionation alone did not appear to further mitigate the interplay effect beyond 6 fractions.

Published reports of pediatric organ motion studies have indicated that such motion is more extensive in adolescents than in younger children and more extensive in the liver and spleen than in the kidneys [22–24]. The present study did not simulate proton therapy directed at the spleen region for many patients because patients requiring such a procedure (eg, those undergoing total lymphatic irradiation) are traditionally treated with anterior-posterior photon fields. Another limitation of this study is that we did not simulate a large number of proton therapy patients. The size of the study cohort was constrained by the number of pediatric patients who had undergone 4D imaging and by the relative lack of heterogeneity in the tumor locations. Nevertheless, interplay effects were quantified in young children with neuroblastoma and in adolescents with diaphragmatic tumors. Until more pediatric-specific data become available, consensus guidelines on implementing pencil-beam scanning proton therapy for adult lung cancer and lymphoma patients [25] may be used, with caution, as starting points for treating pediatric patients with moving tumors. Concerns of particular relevance to the pediatric population, such as obtaining patient cooperation, the effects of sedation on respiration, and issues relating to tumor size and location, should be considered when choosing the optimal mitigation strategies.

As cautioned by Chang et al [25] in the guideline article, the unique tissue heterogeneity in the chest can create significant challenges in predicting accurate pencil-beam scanning dose distribution under the influence of organ or target motion. Monte Carlo dose calculations have been used to improve the accuracy [26, 27], but most interplay effect studies were conducted by using analytical dose calculation algorithms for decreased simulation time. Different from adult lung cancers, moving tumors of pediatric patients are in unique locations such as the retroperitoneal space, anterior mediastinum, chest wall, kidney, spleen, and liver, as included in this study. Because proton beams do not typically go through highly heterogeneous tissues for those sites, we expect the impact of dose calculation algorithms to be smaller and the conclusions stated in this article would remain valid.
